# In vitro sepsis up‐regulates Nociceptin/Orphanin FQ receptor expression and function on human T‐ but not B‐cells

**DOI:** 10.1111/bph.16088

**Published:** 2023-05-11

**Authors:** Mark F. Bird, Christopher P. Hebbes, Anushuya Tamang, Jonathon Mark Willets, Jonathan P. Thompson, Remo Guerrini, Girolamo Calo, David G. Lambert

**Affiliations:** ^1^ Department of Cardiovascular Sciences, Anaesthesia, Critical Care and Pain Management University of Leicester Leicester UK; ^2^ Cellomatics Biosciences Ltd Nottingham UK; ^3^ Molecular and Cell Biology University of Leicester Leicester UK; ^4^ Department of Chemical, Pharmaceutical and Agricultural Sciences University of Ferrara Ferrara Italy; ^5^ Department of Pharmaceutical and Pharmacological Sciences University of Padova Padua Italy

**Keywords:** B‐cells, biosensor, confocal microscopy, Nociceptin/Orphanin FQ, NOP receptor, sepsis, T‐cells

## Abstract

**Background and Purpose:**

In animal models of sepsis, increased activation of the Nociceptin/Orphanin FQ (N/OFQ) receptor NOP is associated with mortality and NOP antagonists improved survival. We have explored the role of the N/OFQ‐NOP system in freshly isolated volunteer human B‐ and T‐cells incubated with lipopolysaccharide (LPS) and peptidoglycan G (PepG) as a model of in vitro sepsis.

**Experimental Approach:**

B‐ and T‐cell NOP expression was measured using the NOP fluorescent probe N/OFQ_ATTO594_, N/OFQ content was measured using immunofluorescence, N/OFQ release was tracked using a CHO_hNOPGαiq5_ biosensor assay and NOP function was measured using transwell migration and cytokine/chemokine release using a 25‐plex assay format. Cells were challenged with LPS/PepG.

**Key Results:**

CD19‐positive B‐cells bound N/OFQ_ATTO594_; they also contain N/OFQ. Stimulation with CXCL13/IL‐4 increased N/OFQ release. N/OFQ trended to reduced migration to CXCL13/IL‐4. Surface NOP expression was unaffected by LPS/PepG, but this treatment increased GM‐CSF release in an N/OFQ sensitive manner. CD3‐positive T‐cells did not bind N/OFQ_ATTO594_; they did contain N/OFQ. Stimulation with CXCL12/IL‐6 increased N/OFQ release. When incubated with LPS/PepG, NOP surface expression was induced leading to N/OFQ_ATTO594_ binding. In LPS/PepG‐treated cells, N/OFQ reduced migration to CXCL12/IL‐6. LPS/PepG increased GM‐CSF release in an N/OFQ sensitive manner.

**Conclusions and Implications:**

We suggest both a constitutive and sepsis‐inducible N/OFQ‐NOP receptor autocrine regulation of B‐ and T‐cell function, respectively. These NOP receptors variably inhibit migration and reduce GM‐CSF release. These data provide mechanistic insights to the detrimental role for increased N/OFQ signalling in sepsis and suggest a potential role for NOP antagonists as treatments.

**Abbreviation**
N/OFQ: nociceptin/orphanin FQ

**What is already known**

- N/OFQ activation of NOP receptors affects immune function.
- N/OFQ is elevated in human and animal sepsis with increased mortality in the latter.

**What does this study add**

- B‐cells constitutively express surface NOP receptors, T‐cells require septic stimulus; N/OFQ is released from both.
- When expressed, N/OFQ activation of NOP receptors variably reduces chemoattractant stimulated migration.

**What is the clinical significance**

- Increased N/OFQ‐NOP receptor signalling in sepsis is detrimental to outcome.
- NOP antagonists are worthy of evaluation for the treatment of sepsis.

## 1 INTRODUCTION

The opioid receptor family comprises the classical μ, δ and κ opioid receptors; these are naloxone sensitive. A fourth member of the opioid receptor family is the naloxone insensitive NOP receptor, which has been given various names including ORL‐1 and OP4. The endogenous ligand for this receptor is the 17 amino acid peptide, Nociceptin/Orphanin FQ (N/OFQ) (Lambert, 2008).

Opioids are best known for their analgesic action but have been implicated as immune modulators; the site(s) of action remain controversial (Al‐Hashimi et al., 2016). We know that μ receptor agonists like morphine depress the immune response, but others with mixed actions like buprenorphine are less effective. We have failed to detect classical opioid receptors on peripheral immune cells, but this is not universally accepted (Eisenstein, 2019; Machelska & Celik, 2020; Plein & Rittner, 2018; Sacerdote, 2006). We believe the target site is likely to be elsewhere. In this regard, there is growing evidence that TLR4 receptors are a peripheral immune cell target for opioids (Giakomidi et al., 2022; Hutchinson et al., 2012).

We have shown that the majority of circulating immune cells express mRNA for the NOP receptor (Al‐Hashimi et al., 2016; Thompson et al., 2013), and using the novel fluorescent probe N/OFQ_ATTO594_ in peripheral blood mononuclear cells, active receptor protein is also detected (Bird et al., 2018; Singh et al., 2016). The immune response is divided into innate and adaptive (although strict separation in not always clear) with the latter relying largely on B‐cell and T‐cell lymphocytes (Brady et al., 2020).

There is evidence for N/OFQ modulation of both B‐ (Anton et al., 2010; Gavioli et al., 2015) and T‐cell (Easten et al., 2009; Waits et al., 2004) function. In isolated (Miltenyi separation) human T‐cells, Waits et al. showed that ultra‐low N/OFQ (10^−14^–10^−^^1^ ^2^M) up‐regulated activation markers and importantly enhanced proliferation measured as [^3^H]thymidine incorporation. Re‐stimulation caused an inhibition of proliferation. In *Staphylococcal enterotoxin* B (SEB) treated cells, there was a significant increase in Prostaglandin D2 (PGD2) secretion. (Waits et al., 2004). In a further study from the same group, Easten and colleagues further examined SEB‐activated human T‐cells (Miltenyi separation). They report again that N/OFQ reduced proliferation in a UFP‐101 (peptide NOP receptor antagonist; Calo et al., 2005) sensitive fashion. This was also reduced when CD80 and CD86 (costimulatory receptors) were blocked (Easten et al., 2009). These data are at variance with our own as these T‐cells displayed NOP receptor mediated function in naïve state. Neither study examined migration.

Sepsis is a life‐threatening host response to infection involving both pro‐ and anti‐inflammatory phases and more specifically is a life‐threatening organ dysfunction caused by a dysregulated host response to infection (Singer et al., 2016). A role for the NOP receptor has been proposed in the pathophysiology of sepsis (Serrano‐Gomez et al., 2011; Thomas et al., 2014). In a rat model of caecal ligation and puncture, NOP receptor activation with N/OFQ increased mortality and NOP receptor antagonism with UFP‐101 improved survival (Carvalho et al., 2008). Moreover, in the intensive care unit, we have shown that N/OFQ is elevated in patients with sepsis and speculate that antagonists might be suitable adjuncts in the treatment of this condition (Thompson et al., 2013). In these studies, the source of endogenous N/OFQ is unknown but presumed to be immune in origin. Evidence in support of this supposition comes from Fiset et al. who showed release of immune reactive‐N/OFQ from human neutrophils (Fiset et al., 2003). To address the issue of N/OFQ release more directly, we recently developed a novel bioassay to track N/OFQ release from single immune cells and showed that a proportion of crude polymorphonuclear leucocytes released this peptide (Bird, Hebbes, et al., 2022).

To further probe the adaptive immune response and make use of our new bioassay in sepsis, we have focussed on freshly isolated human volunteer B‐ and T‐cells. In this paper, we have examined (1) NOP receptor expression in control cells and cells treated with lipopolysaccharide (LPS)/peptidoglycan G (PepG) as an in vitro sepsis mimic and we have deployed a novel fluorescent peptide ligand N/OFQ_ATTO594_, (2) N/OFQ peptide expression, (3) N/OFQ release from single cells, (4) B‐ and T‐cell migration and (5) B‐ and T‐cell cytokine production.

## 2 METHODS

### 2.1 Materials

CHO_hNOPGαiq5_ were supplied by Professor Calò of the University of Padova (originally as a collaboration with Prof T. Costa, Instituto Superiore di Sanita, Rome, Italy). N/OFQ and N/OFQ_ATTO594_ were provided by UFP peptides (University of Ferrara, Italy). PPADS, oATP and SB‐612111 were purchased from TOCRIS (Bristol, UK). Pan B‐ and T‐cell isolation kits were purchased from Miltenyi (Surrey, UK). All Krebs buffer reagents were purchased from Sigma (Gillingham, UK). Cell media was purchased from GIBCO (Paisely, UK). Brilliant violet CD3 (RRID:AB_10962690) and CD19 (RRID:AB_11142678) antibodies were purchased from BioLegend (London, UK). FITC tagged anti‐N/OFQ Ab was from Biorbyt (Cambridge, UK). Cytokine‐25 Plex Kit purchased from ThermoFisher (Loughborough, UK). Transwell chambers (5‐μM pore size) were from Costar (NY, USA). All other reagents were of the highest purity available and generally from Sigma/TOCRIS (Gillingham/Bristol, UK).

### 2.2 B‐ and T‐cell isolation

The University of Leicester (volunteer) ethics committee approved this study, and all volunteers gave written, informed consent. Thirty millilitres of blood were collected into S‐Monovette collection system (Sardstedt, Numbrecht, Germany) containing K‐EDTA (7.5‐ml blood per tube, final EDTA concentration 1.6 mg·ml^−1^). Our donor pool (from the research laboratory) comprised five participants, M:F = 4:1 and age range 25–55. B‐ and T‐cells were separated by either MACSxpress® whole blood pan B‐cell isolation kits or whole blood Pan T‐cell isolation kits (Miltenyi Biotec, Surrey, UK) according to the manufacturer's instructions. For live‐cell bioassay experiments, following extraction of either the pan B‐ or T‐cells, these were washed and suspended in Krebs buffer (126‐mM NaCl, 2.5‐mM KCl, 25‐mM NaHCO_3_, 1.2‐mM NaH_2_PO_4_, 1.2‐mM MgCl_2_, 2.5‐mM CaCl_2_ and 10‐mM HEPES) for counting and imaging. Extractions were carried out at room temperature (RT), and the resulting cell suspension maintained on ice until use. Viability and yields were quantified by Trypan Blue exclusion and counting using a haemocytometer (Bird, Hebbes, et al., 2022). For experiments involving 24‐h LPS (100 ng·ml^−1^)/PepG (20 μg·ml^−1^) treatment, cells were extracted and maintained in RPMI media containing 10% fetal calf serum, streptomycin (100 IU·ml^−1^), penicillin (100 IU·ml^−1^) and amphotericin B (2.5 μg·ml^−1^).

### 2.3 Cell culture

CHO cells transfected with the human NOP receptor and the G‐protein chimera Gα_iq5_, referred to as CHO_hNOPGαiq5_, were maintained in Hams F12:DMEM (Invitrogen, Paisley, UK) and were supplemented with 10% fetal calf serum, streptomycin (100 IU·ml^−1^), penicillin (100 IU·ml^−1^), amphotericin B (2.5 μg·ml^−1^) G‐418 (200 μg·ml^−^^1^) and Hygromycin (100 μg·ml^−^^1^). In experimental sepsis models, B‐ or T‐cells were maintained in RPMI which contained both 100 ng·ml^−1^ LPS and 20 μg·ml^−1^ PepG for 24 h. Cells were maintained in 5% CO_2_ humidified air at 37°C, with CHO_hNOPGαiq5_ subcultured routinely after reaching ≥90% confluency (Bird, Hebbes, et al., 2022).

### 2.4 Confocal microscopy

#### 2.4.1 NOP receptor and N/OFQ expression

Investigation of NOP receptor surface expression in live B‐ or T‐cells was performed using cells plated onto ethanol sterilised 28‐mm Menzel glaser #1 coverslips (Thermo Scientific, UK), which were pretreated with Celltak™ (1 μg·ml^−1^; Sigma Aldrich, UK), for 24 h. Brilliant Violet™ 421 nm tagged CD19 (Pan B‐cell marker) or Brilliant Violet™ 421 nm CD3 (pan T‐cell marker) antibodies (1:1000; Biolegend, UK) were used as cell markers. Cells were maintained in RPMI media as mentioned previously for 24 h, with half the sample incubated with 100 ng·ml^−1^ LPS and 20 μg·ml^−1^ PepG. Following this, B‐ or T‐cell containing coverslips were incubated at 4°C in Krebs buffer, pH 7.4, and 100 nM of the fluorescent ligand N/OFQ_ATTO594_ was added and allowed to incubate for 5 min. Excess N/OFQ_ATTO594_ was washed off and images were captured on a Nikon C1Si microscope (Nikon, UK) using a 60× immersion oil objective. B‐ or T‐cells were imaged using a 594‐nm wavelength laser and images collected using 620‐nm filter screening on C1Si software. As N/OFQ_ATTO594_ is an agonist, incubations needed to be performed at 4°C to prevent internalisation (Bird, Hebbes, et al., 2022). To confirm NOP receptor interaction, the NOP receptor antagonist SB‐612111 (10 μM) was included to define any non‐specific binding (Spagnolo et al., 2007; Zaratin et al., 2004).

Immunofluorescence was used to determine the presence of N/OFQ peptide in B‐ or T‐cells and to overlay this with NOP receptor expression. B‐ and T‐cells were collected from whole blood as above and incubated on high‐precision microscope cover glasses (no 1.5H) coated with Celltak™ (1 μg·ml^−1^) for 24 h in RPMI media. Following incubation, cells were covered in 4% paraformaldehyde and allowed to fix for 15 min at RT. Following incubation, cells were washed for 3 × 5 min periods in phosphate buffered saline (PBS). Cells were then blocked in phosphate buffered saline (PBS) with 5% fetal calf serum and 0.3% Triton™ X‐100 for 1 h at RT. This (blocking) buffer was aspirated, and then a dilution buffer (PBS, 1%BSA, 0.3% Triton™ X‐100) containing FITC‐tagged anti‐N/OFQ Ab (Biorbyt, Cambridge; 1:1000) and either Brilliant Violet™ 421 nm tagged CD19 (Pan B‐cell marker) or Brilliant Violet™ 421 nm CD3 (pan T‐cell marker) antibodies (1:1000) (Biolegend, UK) was added to cover glasses. Cells were left to incubate overnight at 4°C, following which, cells were rinsed three times in PBS and dilution buffer containing 100 nM N/OFQ_ATTO594_ was added and left to incubate for 24 h at 4°C. Cells were again washed and mounted onto Surgipath pre‐cleaned microslides (Leica, UK) with Prolong™ Glass antifade mountant. Cells were imaged sequentially (2 s per frame) at 594 nm (RED‐N/OFQ_ATTO594_), 488 nm (GREEN‐ Anti‐N/OFQ Ab) and 420 nm (BLUE‐ CD markers) using C1Si confocal microscope with 60× oil immersion objective and Nikon C1Si software to collect images.

The immuno‐related procedures used in this study comply with the recommendations made by the *British Journal of Pharmacology* (Alexander et al., 2018).

#### 2.4.2 Live cell release of N/OFQ

This was largely as described in Bird, Hebbes, et al. (2022). CHO_hNOPGαiq5_ (biosensor) cells were seeded onto ethanol sterilised 28‐mm Menzel glaser #1 coverslips and incubated for 24 h in F12:DMEM. When required for use, biosensor cells were incubated with 6‐μM FLUO‐4AM calcium dye for 30 min at RT in the dark. Isolated B‐cells and T‐cells were incubated for 15 min with 6‐μM FLUO‐4AM to dye load prior to injection onto FLUO‐4AM loaded CHO_hNOPGαiq5_ biosensor cells.

Following FLUO‐4AM incubation, cells were transferred to a PDMI‐2 micro‐incubator (Harvard Apparatus, USA) and perfused with 37°C Krebs buffer. Following washing, cells were incubated with both 5‐mM pyridoxalphosphate‐6‐azophenyl‐2′,4′‐disulfonic acid (PPADS) and 0.8‐mM adenosine 5′‐triphosphate‐2′,3′‐dialdehyde (oATP) to block endogenous purinergic receptors in the biosensor cells; this will prevent any released ATP from influencing the signal as the biosensor cells express endogenous purinergic receptors (Bird, Hebbes, et al., 2022). To confirm NOP receptor activation, the NOP receptor antagonist SB‐612111 (10 μM) was included (Spagnolo et al., 2007; Zaratin et al., 2004). Isolated B‐ or T‐cells were maintained in 37°C Krebs immediately after isolation. B‐ or T‐cells were allowed to settle following which they were stimulated with either 1 μg·ml^−1^ CXCL13 and IL‐4 (B‐cell activation; Carlsen et al., 2002; Mori et al., 2000) or by 1 μg·ml^−1^ CXCL12 and IL‐6 (T‐cell activation; Ticchioni et al., 2002; Weissenbach et al., 2004). Increased Ca^2+^ in CHO_hNOPGαiq5_ and B‐ and T‐cells was measured via a 488 nm laser, and emission was recorded at 513–556 nm. Time series were recorded at 2‐s intervals. Gain and laser power were determined empirically and maintained constant. A positive response was conservatively defined as in Bird, Hebbes, et al. (2022) as increased relative fluorescence ≥1.8 over baseline, with baseline defined as previously described in Bird, Hebbes, et al. (2022). To determine relative fluorescence, regions of interest were defined by mapping single CHO_hNOPGαiq5_ cells and measuring changes in fluorescence levels compared with basal activity.

### 2.5 Cell chemotaxis

B‐ or T‐cells were isolated and incubated with 10‐nM Celltracker Green BODIPY dye (ThermoFisher, UK) for 24 h in RPMI alone or RPMI containing 100 ng·ml^−1^ LPS and 20 μg·ml^−1^ PepG. Following 24‐h incubation, cells were counted, and 200,000 cells were added per experimental well. Wells contained 12‐well transwell chambers (5‐μm pore size, Costar NY). All wells, barring one per donor to measure unstimulated chemotaxis, contained either CXCL13 and IL‐4 (B‐cell chemoattractants) (Carlsen et al., 2002; Mori et al., 2000) or CXCL12 and IL‐6 (T‐cell chemoattractants) (Ticchioni et al., 2002; Weissenbach et al., 2004) for 4 h at 37°C. Samples (both untreated and LPS/PepG stimulated) were measured alone, with 100 nM N/OFQ, with a combination of 1‐μM N/OFQ and 10‐μM SB‐61211 or 10‐μM SB‐61211 alone. Following experimental incubation, transwells were removed, and cell fluorescence (of the migrated cells) was counted in a TriStar^2^S plate reader (Berthold, UK) (excitation: 525 nm; emission: 488 nm). Cell migration was measured as a percentage compared to control fluorescence.

### 2.6 Cytokine and chemokine release

B‐ or T‐cells were isolated, counted and distributed at 200,000 cells per well in six‐well plates containing either RPMI alone or in RPMI containing 100 ng·ml^−1^ LPS and 20 μg·ml^−1^ PepG for 24 h. During this incubation period, LPS/PepG treated wells were co‐stimulated with 100‐nM N/OFQ. Following treatment, media was collected, centrifuged (1500 rpm, 5 min at 4°C) and supernatant collected. A cytokine 25‐plex human panel kit (Thermo Fisher, UK) was used to test for cytokine and chemokine release using a Luminex MAGPix™ detection system. The kit measures the following cytokines: GM‐CSF, eotaxin, IFN‐α, IP‐10, IFN‐γ, MCP‐1, IL‐1β, MIG, IL‐1RA, MIP‐1α, IL‐2, MIP‐1β, IL‐2R, RANTES, IL‐4, IL‐5, IL‐6, IL‐7, IL‐8, IL‐10, IL‐12 (p40/p70), IL‐13, IL‐15, IL‐17 and TNF‐α (Thermofisher, UK).

### 2.7 Study design and data analysis

The data and statistical analysis comply with the recommendations of the *British Journal of Pharmacology* on experimental design and analysis in pharmacology (Curtis et al., 2022). Where mean data are used, this is ±SEM, and analysis was performed in GraphPad Prism V7; all groups are evenly matched with no missing values and driven by both power calculation (below) and volunteer numbers at n = 5. Each volunteer is one experiment. Appropriate statistical testing is described in figure legends and *P* < 0.05 was considered significant. Where data are normalised to control, this is for presentation only with analysis performed on the raw data. Specimen confocal data sets are presented, and all confocal images were analysed using ImageJ (Version 1.53s). Changes in fluorescent intensity are measured as previously described in Bird, Hebbes, et al. (2022) where regions of interest of increased intensity in CHO_hNOPgαqi5_ cells are superimposed onto initial image and used to measure the mean increase in fluorescent intensity. N/OFQ_ATTO594_ binding and immunofluorescence for N/OFQ peptide were present or absent with numbers n = 5 (our volunteer pool), and images were verified by the senior author. Experiments using N/OFQ_ATTO594_ was ‘real time’ and not blinded. Release of N/OFQ using CHO_hNOPGαiq5_ (biosensor) cells was real time and not blinded. Chemotaxis experiments were powered on B‐cell migration data (Blink et al., 2005) with power 80% at *P* < 0.05 to detect a 30% decrease in migration compared to control group (same volunteer); this yielded n = 3 per group, which was increased to a final sample size of 5. Cytokine experiments were powered on TNF‐α data (Damas et al., 1989) with power 80% at *P* < 0.05 to detect a 30% increase in release compared to control group (same volunteer); this yielded n = 3 per group, which was increased to a final sample size of 5. These experiments were not blinded, but the final data spreadsheet from the instrument was provided for analysis independent of loading the machine.

### 2.8 Nomenclature of targets and ligands

Key protein targets and ligands in this article are hyperlinked to corresponding entries in http://www.guidetopharmacology.org, and are permanently archived in the Concise Guide to PHARMACOLOGY 2021/22 (Alexander Christopoulos et al., 2021; Alexander Fabbro et al., 2021).

## 3 RESULTS

### 3.1 B‐cells

**FIGURE 1:**
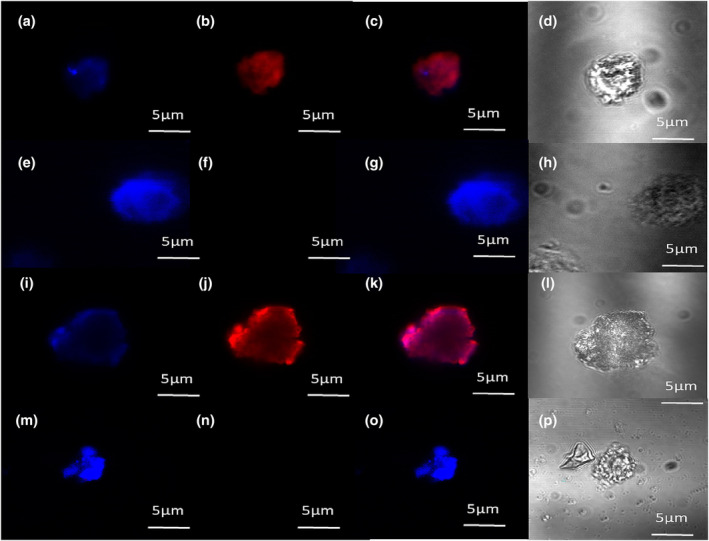
B‐cell expression of NOP. Live B‐cells were incubated for 24 h in either control medium or medium containing 100 ng·ml^−1^ LPS and 20 μg·ml^−1^ PepG. In control samples, the pan B‐cell marker, CD19 (blue), bounds to the cell surface (a) as did 100‐nM fluorescent peptide N/OFQ_ATTO594_ (red) (b), with overlay shown in (c) and transmitted light image shown in (d). To determine selectivity of N/OFQ_ATTO594_ binding, B‐cells were pre‐treated with 10 μM of the NOP antagonist, SB‐612111. These cells were incubated with CD19 (e) and 100 nM N/OFQ added, which failed to bind (f) with overlay shown in (g) and transmitted light image shown in (h). B‐cells treated for 24 h with 100 ng·ml^−1^ LPS and 20 μg·ml^−1^ PepG showed similar binding of CD19 (i) as well as binding of N/OFQ_ATTO594_ (j), with composite shown in (k) and transmitted light image shown in (l). LPS/PepG treated B‐cells (m) were pretreated with 10‐μM SB‐612111, following which they were treated with 100‐nM N/OFQ_ATTO594_, which did not bind to the cell surface (n). Composite image is shown in (o) and transmitted light image shown in (p). Images are representative of five separate experiments from separate donors.

Isolated live B‐cells were probed with the NOP‐selective fluorescent ligand N/OFQ_ATTO594_ to detect the presence of NOP receptors on the cell surface in untreated and LPS (100 ng·ml^−1^)/PepG (20 μg·ml^−1^) treated cells (A high expression CHO_hNOP_ image is shown in Figure S1 for comparison). The pan B‐cell marker, CD19 (blue), was used to identify B‐cells (Figure 1a), following which 100‐nM N/OFQ_ATTO594_ (red) was added (Figure 1b), which bound to the cell surface. Composite image is shown in Figure 1c and transmitted light image of live B‐cell shown in Figure 1d. To determine binding selectivity, the competitive NOP receptor antagonist SB‐612111 (10 μM) was preincubated on CD19 stained B‐cells (Figure 1e) before addition of 100‐nM N/OFQ_ATTO594_ (Figure 1f), which failed to bind in these conditions; composite image is shown in Figure 1g and transmitted light of live B‐cell shown in Figure 1h. In LPS/PepG treated B‐cells (Figure 1i), N/OFQ_ATTO594_ bound to the B‐cell surface (Figure 1j). Composite image of CD19 and N/OFQ_ATTO594_ binding is shown in Figure 1k and transmitted light image of LPS/PepG treated B‐cell shown in Figure 1l; in order to determine specific binding of N/OFQ_ATTO594_ in LPS/PepG treated B‐cells, CD19 labelled B‐cells were preincubated with 10‐μM SB‐612111 (Figure 1m), following which 100‐nM N/OFQ was added (Figure 1n) demonstrating no binding on the receptor surface. A composite image of the B‐cell is shown in Figure 1o and transmitted light image of the live B‐cell shown in Figure 1p. That the imaging experiments were performed at 4°C, and that non membrane permeant SB‐612111 fully prevented probe binding, confirm binding of the probe to the cell surface. There was a predicted and marked LPS/PepG driven change in B‐cell shape (Gustave et al., 2018).

**FIGURE 2:**
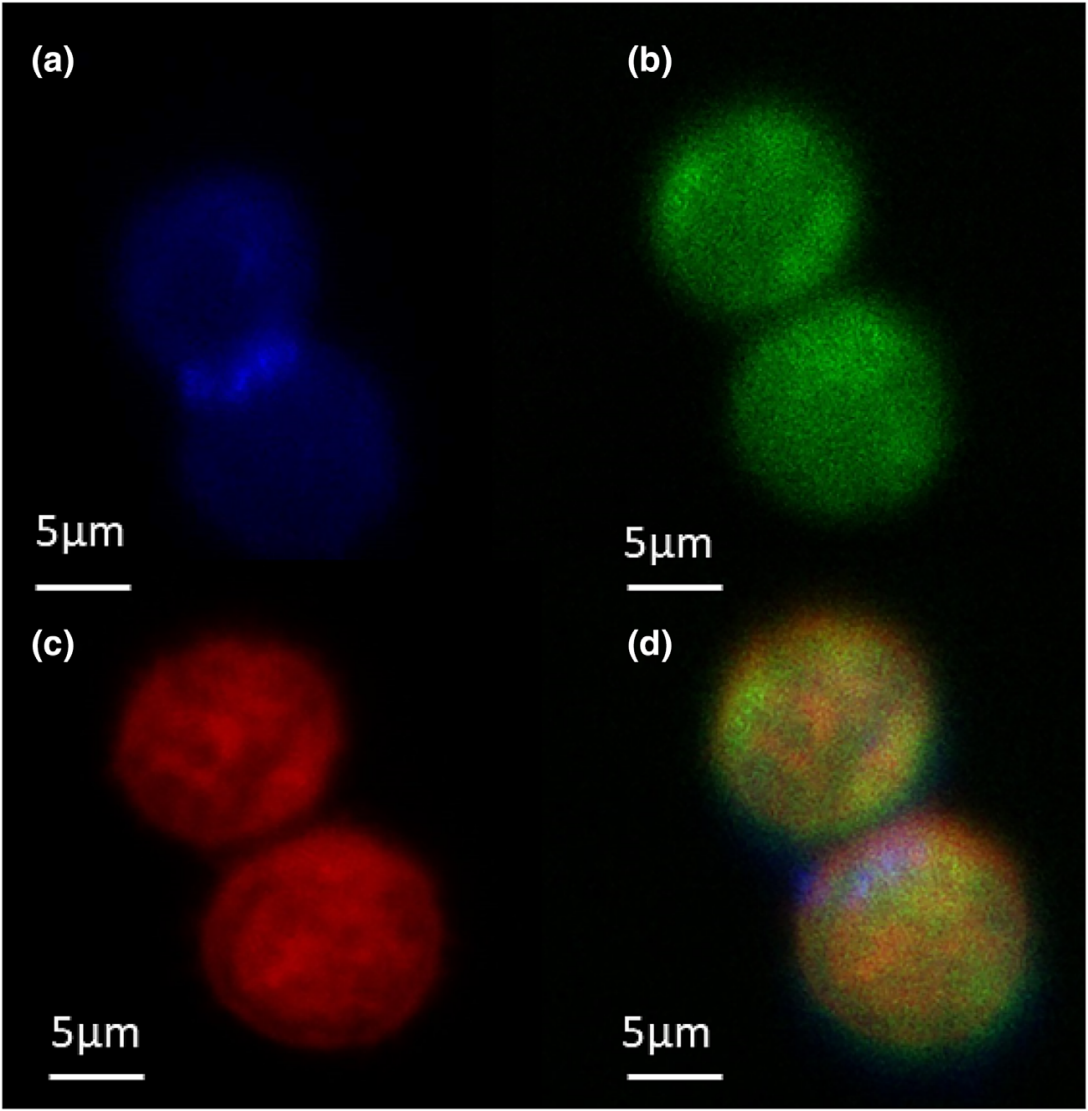
Determination of B‐cell N/OFQ and NOP expression in **permeabilised** **and fixed** cells. Immunofluorescence assays on CD19 (blue) stained B‐cells (a) show the presence of N/OFQ (green) (b) and the NOP receptor (c) (via N/OFQ_ATTO594_‐red). The composite image is shown in (d). Images are representative of five separate experiments from separate donors.

To determine the presence of N/OFQ peptide in B‐cells, immunofluorescence experiments were performed in permeable (access to intracellular pools), fixed cells (Figure 2). B‐cells were again identified with CD19 (Figure 2a, blue) and stained with anti‐N/OFQ antibody (Figure 2b, green). Presence of NOP receptor was further confirmed by addition of 100‐nM N/OFQ_ATTO594_ (Figure 2c, red). Composite image shows diffuse spread of both NOP and N/OFQ throughout the cell (Figure 2d). Antibody binding and SB‐612111 controls are shown in Figure S2.

**FIGURE 3:**
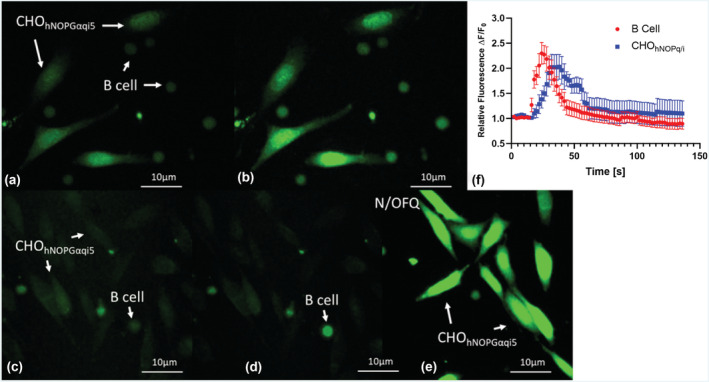
Biosensor measurement of N/OFQ release from B‐cells. (a) B‐cells were plated onto coverslips with FLUO4‐AM loaded CHO_hNOPGαiq5_ biosensor cells preincubated with PPADS (5 mM) and oATP (0.8 mM) (Bird, Hebbes, et al., 2022). (b) B‐cells were stimulated by CXCL13 and IL‐4, and increases in calcium were measured in both B‐cells and CHO_hNOPgqi5_ cells. To ensure activation of CHO_hNOPGαiq5_ biosensor cells was due to release of N/OFQ, experiments were performed in the presence of 10‐μM SB‐612111 (c). (d) Stimulation of B‐cells with CXCL13 and IL‐4 increased Ca^2+^ but failed to produce a response in FLUO4‐AM loaded CHO_hNOPGαiq5_ biosensor cells. (e) Following washing, cells were retested with 100‐nM N/OFQ to assess biosensor cell activity and viability. (f) Mean (SEM) time courses from five responding B‐cells–biosensor cells as representatives from each individual donor. B‐cell activation leads to activation of CHO_hNOPGαiq5_ biosensor cells. Images are also representative of five separate experiments from separate donors. Over these five donors and for cells in plane, all B‐cells responded to CXCL13 and IL‐4, and of 18 biosensor cells with a B‐cell in proximity, an average of 95% responded.

A major aim of this work was to determine whether B‐ and T‐cells release N/OFQ. Using a previously described bioassay (Bird, Hebbes, et al., 2022), CHO_hNOPGαiq5_ biosensor and immune cells, loaded with the calcium dye FLUO4‐AM in the presence of the purinergic antagonists PPADS and oATP, were used to detect the release of N/OFQ (Figure 3). B‐cells (also incubated with FLUO‐4AM for 15 min prior to adding) were added onto a coverslip containing CHO_hNOPGαiq5_ biosensor cells (Figure 3a) and stimulated with CXCL13 and IL‐4, which in turn leads to an increase in Ca^2+^ in B‐cells and then CHO_hNOPGαiq5_ biosensor cells (Figure 3b,f). To confirm that activation of CHO_hNOPGαiq5_ biosensor cells was due to released N/OFQ, cells were pre‐incubated with the NOP antagonist 10‐μM SB‐612111 (Figure 3c). B‐cells were added onto the coverslip and stimulated with by CXCL13 and IL‐4 and, in the presence of SB‐612111, failed to increase Ca^2+^ (Figure 3d) in the biosensor cells, but there was an increase in the B‐cells. Cells were washed free of antagonist, and 100‐nM N/OFQ was added to confirm CHO_hNOPGαiq5_ biosensor cells viability (Figure 3e). Collectively, these data indicate that B‐cells contain and release N/OFQ peptide.

**FIGURE 4:**
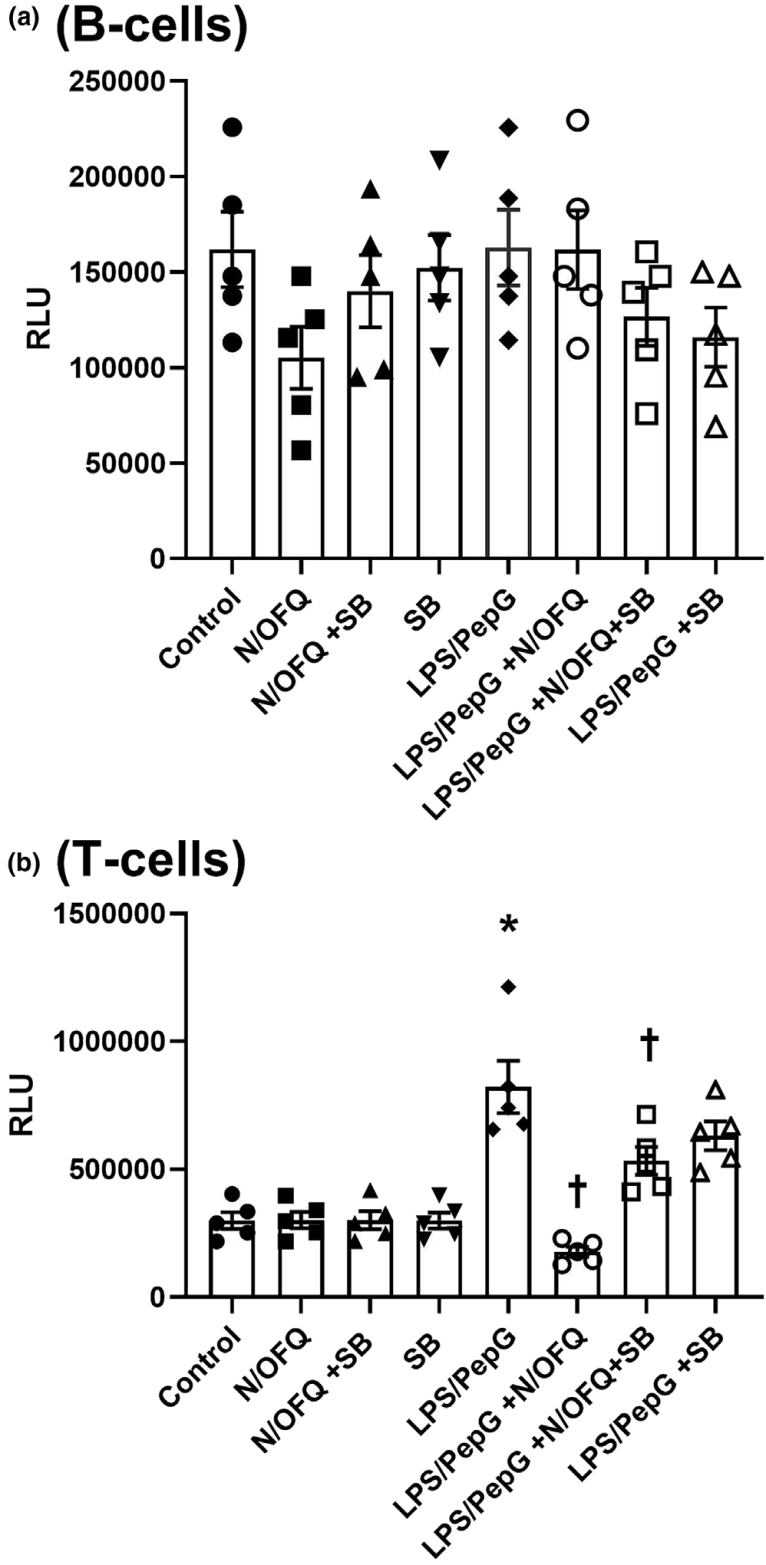
(a) B‐cell migration and (b) T‐cell migration. In both cell types, 100‐nM N/OFQ, 10‐μM SB‐612111 and 100 ng·ml^−1^ LPS plus 20 μg·ml^−1^ PepG treatment was used. Data are the mean (±SEM) from five separate donors. Analysis was carried out employing two‐way ANOVA (B‐cells: there was no overall effect of LPS, *P* = 0.87; T‐cells: there was a significant effect of LPS, *P* < 0.0001) and Sidak post hoc analysis. In (b), * statistically significant increase compared with untreated control (*P* < 0.05). ^**+**^lower than LPS/PepG induced migration activity (*P* < 0.05).

To explore whether N/OFQ‐NOP regulates the B‐cell function, migration was measured using the fluorescent marker Bodipy and the chemoattractants CXCL13 and IL‐4 under control conditions and with LPS (100 ng·ml^−1^)/PepG (20 μg·ml^−1^) as a mimic of in vitro sepsis. In control conditions, N/OFQ reduced B‐cell migration compared with control (by two‐way ANOVA, this did not reach significance). This was reversed by 10‐μM SB‐612111. The addition of 10‐μM SB‐612111 alone had no effect on B‐cell migration in control samples. In B‐cells treated with LPS/PepG for 24 h, N/OFQ had no effect on migration, whereas incubation with SB‐612111, both in conjunction with N/OFQ and alone, tended towards reduced migration (by two‐way ANOVA, this did not reach significance) (Figure 4a).

**FIGURE 5:**
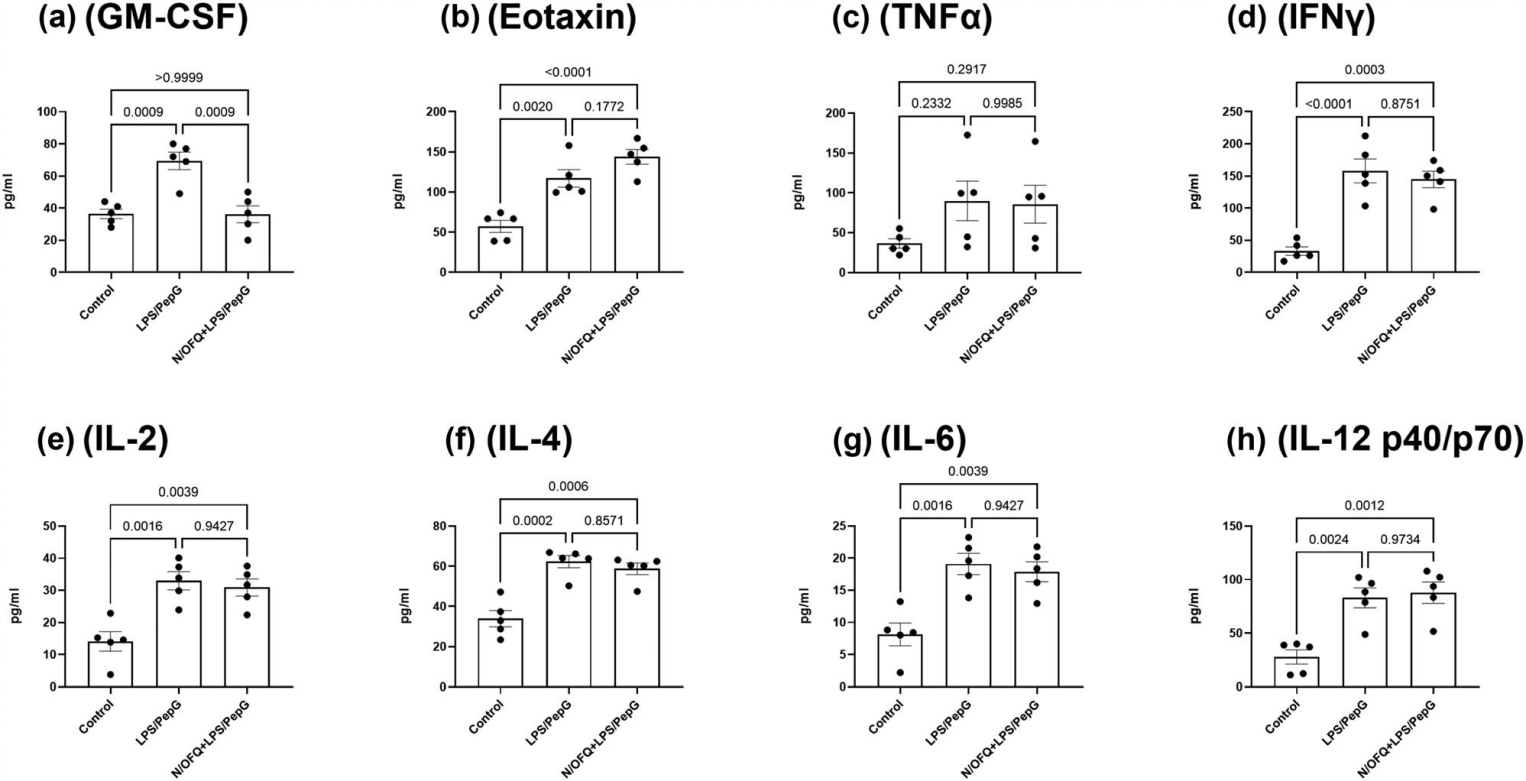
Cytokine/Chemokine release from B‐cells. N/OFQ (100 nM) and 100 ng·ml^−1^ LPS plus 20 μg·ml^−1^ PepG treatment were used. Data are the mean (±SEM) from five separate donors. Statistical analysis was carried out using ANOVA. GM‐CST: *F*(2, 12) = 16.67:*P* = 0.0003; eotaxin: *F*(2, 12) = 22.88:*P* < 0.0001; TNF‐α: *F*(2, 12) = 2.186:*P* = 0.155; IFN‐γ: *F*(2, 12) = 25.45:*P* < 0.0001; IL‐2: *F*(2, 12) = 13.2–:*P* = 0.0009; IL‐4: *F*(2, 12) = 21.12:*P* < 0.0001; IL‐6: *F*(2, 12) = 13.20:*P* = 0.0009; IL‐12 (p40/p70): *F*(2, 12) = 14.44:*P* = 0.0006. Exact statistical significance (Sidak post hoc analysis) is shown.

B‐cell activity was further studied by assessing cytokine/chemokine release (Figure 5). Of the 25 measured, eight reached the threshold for detection in the 25‐cytokine/chemokine panel kit. All eight (GM‐CSF, eotaxin, TNF‐α, IFN‐γ, IL‐2, IL‐4, IL‐6 and IL‐12 [p40/p70]) showed increased secretion in the media following 24‐h LPS (100 ng·ml^−1^)/PepG (20 μg·ml^−1^) treatment. Increased concentrations of all but one (GM‐CSF) were maintained when 24‐h LPS/PepG treatment was combined with 100‐nM N/OFQ. GM‐CSF returned to basal levels after incubation with N/OFQ.

### 3.2 T‐cells

**FIGURE 6:**
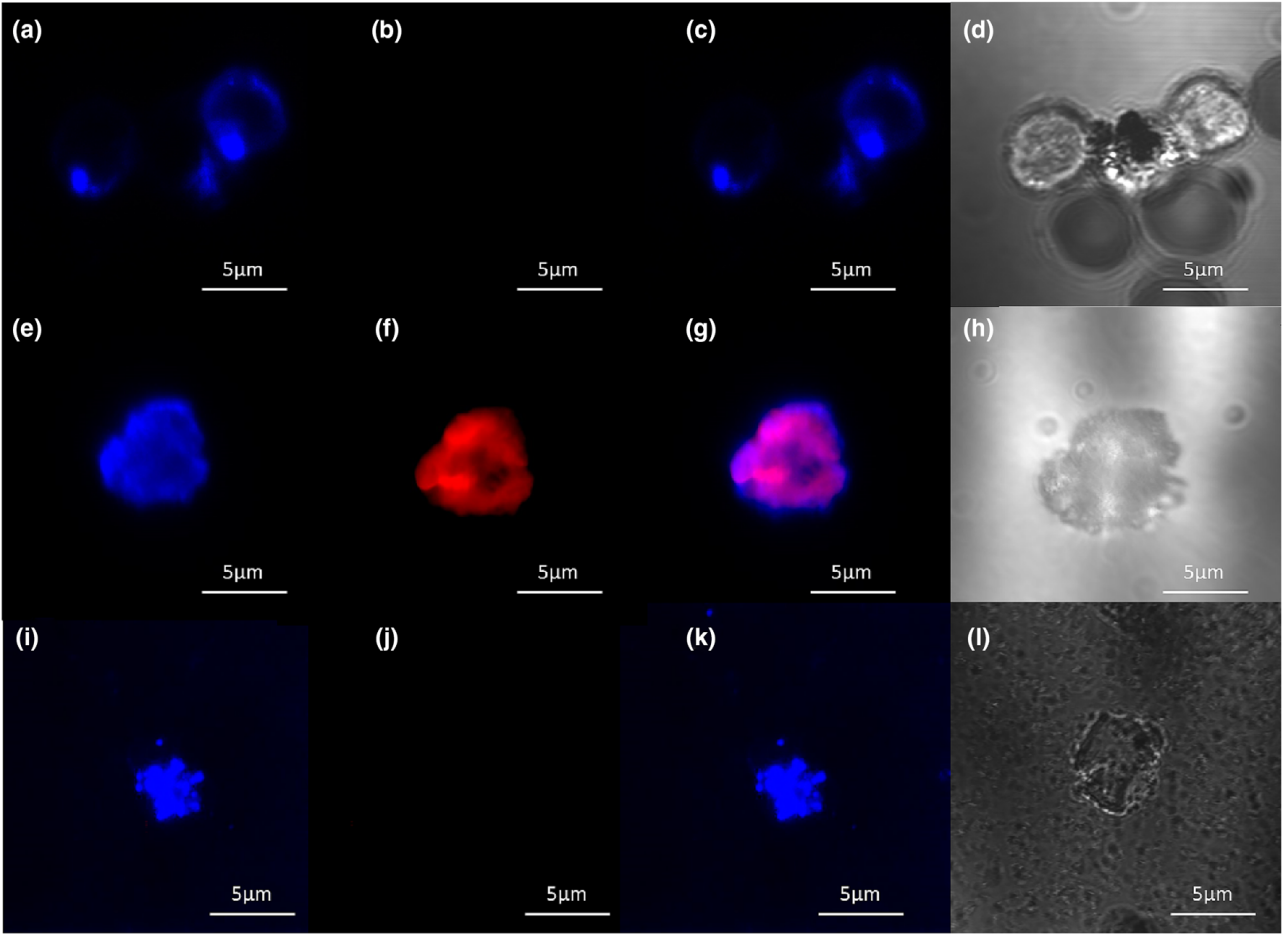
T‐cell expression of NOP. Live T‐cells were incubated for 24 h in either control medium or medium containing 100 ng·ml^−1^ LPS and 20 μg·ml^−1^ PepG. In control samples, the pan T‐cell marker, CD3 (blue), bound to the cell surface (a); however, 100‐nM fluorescent peptide N/OFQ_ATTO594_ (red) failed to bind to the cell surface (b). Overlay is shown in (c) with transmitted light image shown in (d). T‐cells treated for 24 h with LPS/PepG showed similar binding of CD3 (e) as well as binding of N/OFQ_ATTO594_ (f), with overlay shown in (g) and transmitted light shown in (h). To confirm N/OFQ_ATTO594_ binding selectivity in LPS/PepG treated T‐cells, these were pre‐treated with 10‐μM SB‐612111. CD19 binding was demonstrated (i) but 100‐nM N/OFQ failed to bind (j). Overlay is shown in (k) with transmitted light image shown in (l). Images are representative of five separate experiments from separate donors.

Isolated live T‐cells were used in confocal microscopy experiments with anti‐CD3 Ab (blue) to identify T‐cells and 100‐nM N/OFQ_ATTO594_ (red) to identify NOP (Figure 6). In control experiments, T‐cells were positively stained with CD3 (Figure 6a), but N/OFQ_ATTO594_ failed to bind to the cell surface (Figure 6b); composite image is shown in Figure 6c and transmitted light image of live T‐cell shown in Figure 6d. Following 24‐h treatment with LPS (100 ng·ml^−1^)/PepG (20 μg·ml^−1^), CD3‐positive T‐cells (Figure 6e) bound 100‐nM N/OFQ_ATTO594_ (Figure 6f), with composite image showing colour overlap (Figure 6g) and transmitted light image of live T‐cell shown in Figure 6h. To confirm binding selectivity of N/OFQ_ATTO594_ to NOP, LPS/PepG treated T‐cells were pre‐incubated with 10‐μM SB‐612111 (Figure 6i), following which they were treated with 100‐nM N/OFQ_ATTO594_ (Figure 6j), with N/OFQ_ATTO594_ failing to bind. Composite image is shown in Figure 6k and transmitted light image of live T‐cell shown in Figure 6l. That the imaging experiments were performed at 4°C and that non membrane permeant SB‐612111 fully prevented probe binding confirm binding of the probe to the cell surface. T‐cell shape changed markedly with LPS/PepG treatment.

**FIGURE 7:**
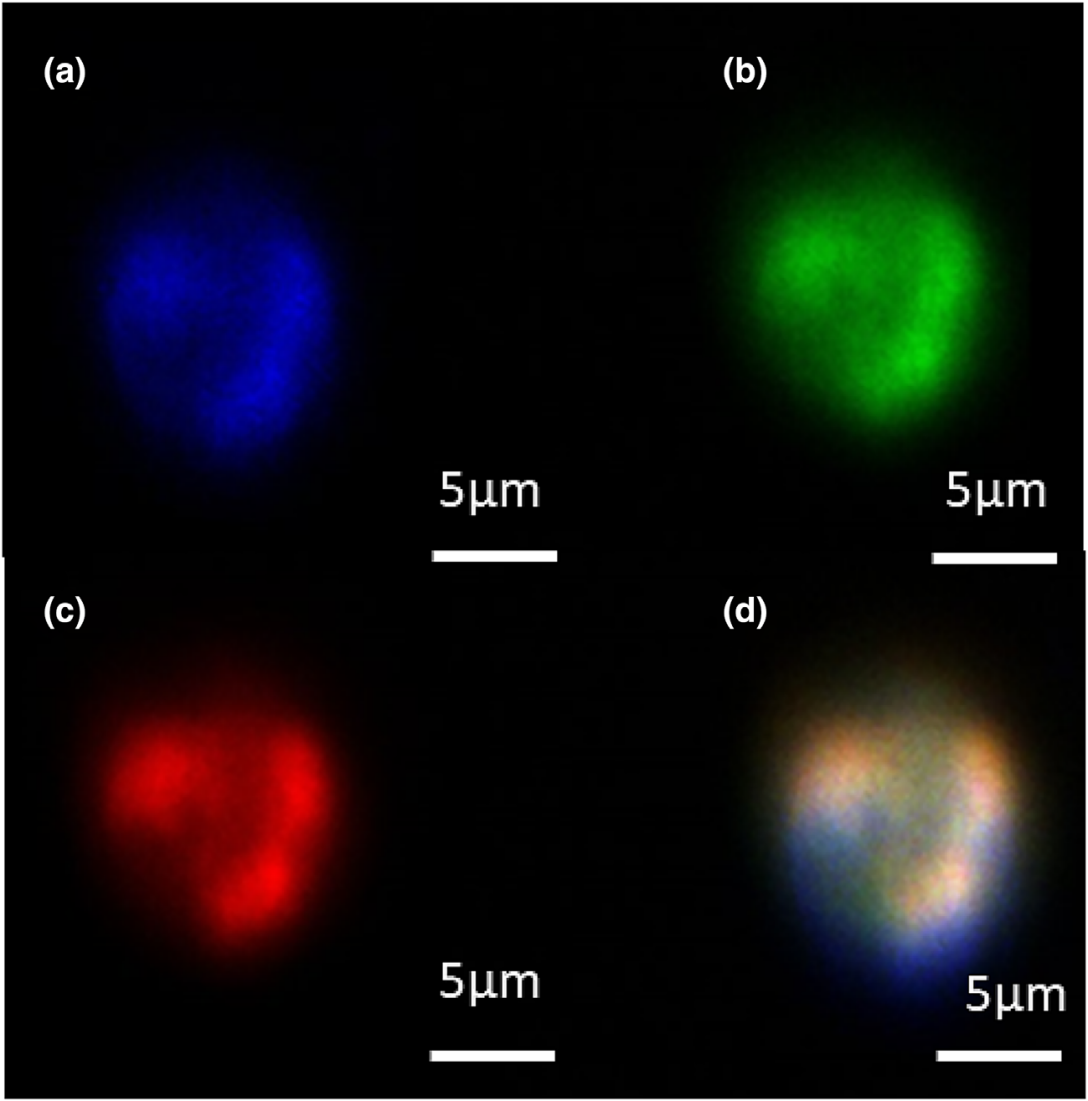
Determination of T‐cell N/OFQ and NOP expression in **permeabilised and fixed** cells. Immunofluorescence assays on CD3 (blue) stained T‐cells (a) show the presence of N/OFQ (green) (b) and the NOP receptor (c) (via N/OFQ_ATTO594_‐red). The composite image is shown in (d). Images are representative of five separate experiments from separate donors.

To determine T‐cell N/OFQ peptide expression, permeabilised (access to intracellular pools), fixed and anti‐CD3 antibody stained cells (Figure 7a, blue) were used in immunofluorescence experiments. In these cells, anti‐N/OFQ Ab binding was detected (Figure 7b, green) in addition to the binding of 100 nM N/OFQ_ATTO594_ (Figure 7c, red). A composite image shows diffuse spread of both N/OFQ and NOP throughout the cell, Figure 7d. Antibody binding and SB‐612111 controls are shown in Figure S3.

**FIGURE 8:**
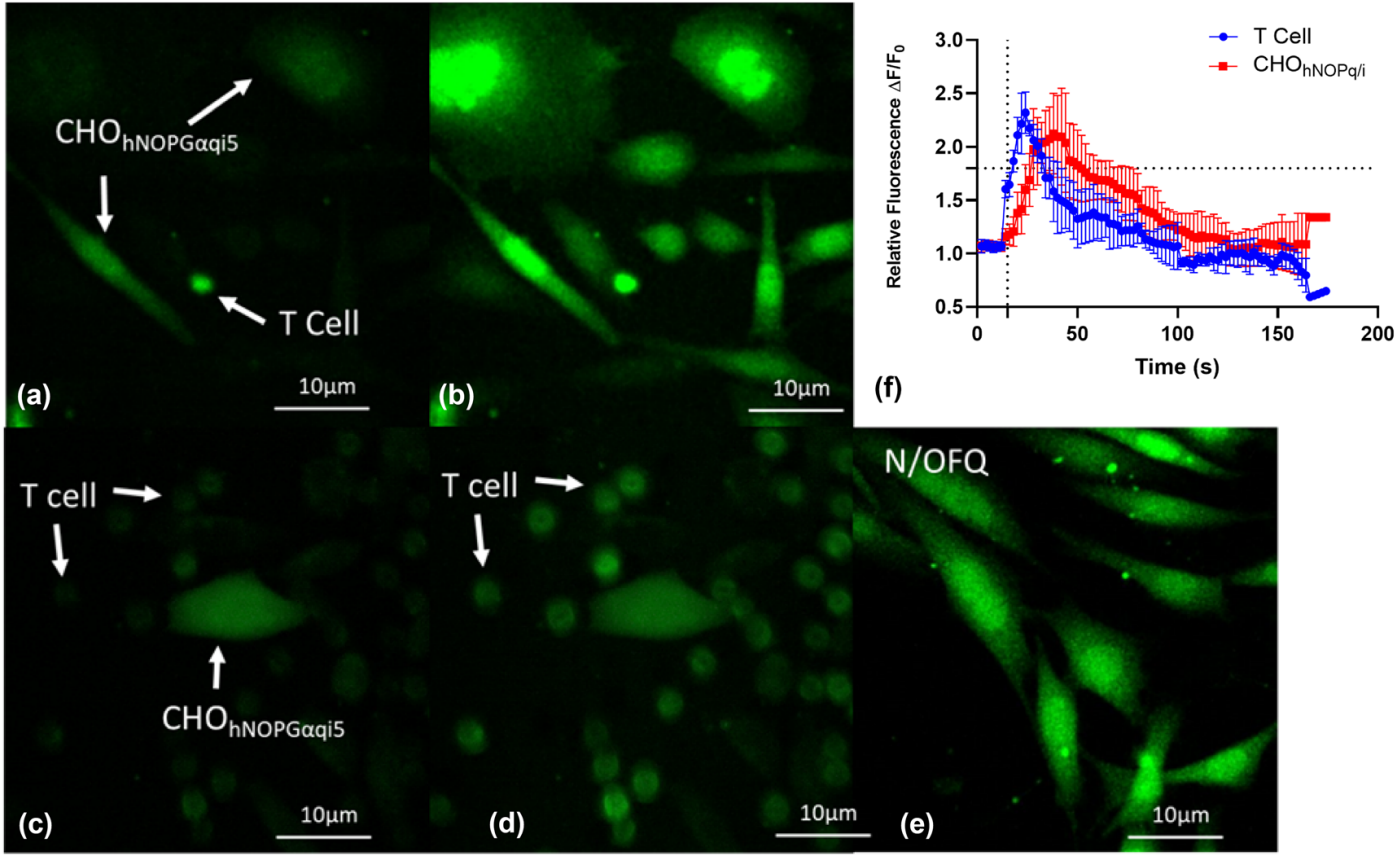
Biosensor measurement of N/OFQ release from T‐cells. (a) T‐cells were plated onto coverslips with FLUO4‐AM loaded CHO_hNOPGαiq5_ biosensor cells preincubated with PPADS (5 mM) and oATP (0.8 mM) (Bird, Hebbes, et al., 2022). (b) T‐cells were stimulated by CXCL12 and IL‐6, and increases in calcium were measured in both T‐cells and CHO_hNOPgqi5_ cells. To ensure activation of CHO_hNOPGαiq5_ biosensor cells was due to release of N/OFQ, experiments were performed in the presence of 10‐μM SB‐612111 (c). (d) Stimulation of T‐cells with CXCL12 and IL‐6 increased Ca^2+^ but failed to produce a response in FLUO4‐AM loaded CHO_hNOPGαiq5_ biosensor cells. (e) Following washing, cells were retested with 100‐nM N/OFQ to assess biosensor cell activity and viability. (f) Mean (SEM) time courses from five responding T‐cells–biosensor cells as representatives from each individual donor. T‐cell activation leads to activation of CHO_hNOPGαiq5_ biosensor cells. Images are also representative of five separate experiments from separate donors. Over these five donors and for cells in plane, all T‐cells responded to CXCL12 and IL‐6, and of 15 biosensor cells with a T‐cell in proximity, an average of 82% responded.

T‐cell release of N/OFQ was tested in the previously described CHO_hNOPGαiq5_ biosensor cells loaded with FLUO‐4 (Figure 8). T‐cells (also loaded with FLUO‐4AM for 15 min prior to addition) were added onto coverslips containing CHO_hNOPGαiq5_ biosensor cells pretreated with PPADS and oATP and allowed to settle (Figure 8a). T‐cells were stimulated with CXCL12/IL‐6 following which increases in fluorescence induced by calcium release were measured in both T‐ and biosensor cells (Figure 8b,f). To determine whether increases in fluorescence in CHO_hNOPGαiq5_ biosensor cells were induced by N/OFQ, cells were preincubated with 10‐μM SB‐612111 (Figure 8c), following which T‐cell were activated with CXCL12/IL‐6 (Figure 8d); this failed to produce increases in fluorescence in CHO_hNOPGαiq5_ biosensor cells, but there was an increase in the T‐cells. CHO_hNOPGαiq5_ cell responsiveness was confirmed by washing and re‐adding 100‐nM N/OFQ (Figure 8e); there was an increase in fluorescence.

T‐cell migration was measured as noted above with CXCL12 and IL‐6 as chemoattractants (Figure 4b). In control cells, the addition of 100‐nM N/OFQ and/or 10‐μM SB‐612111 did not alter T‐cell migration towards the chemoattractant; this is consistent with a lack of cell surface NOP receptors. When treated with LPS (100 ng·ml^−1^)/PepG (20 μg·ml^−1^), T‐cell migration increased when compared with control activity. When 100‐nM N/OFQ is added to LPS/PepG treated cells, migration is inhibited. The addition of 10‐μM SB‐612111 reverses N/OFQ‐induced inhibition in LPS/PepG treated cells; this did not fully return LPS/PepG treated levels. SB‐612111 alone produced a reduction in LPS/PepG stimulated migration (by two‐way ANOVA, this did not reach significance).

**FIGURE 9:**
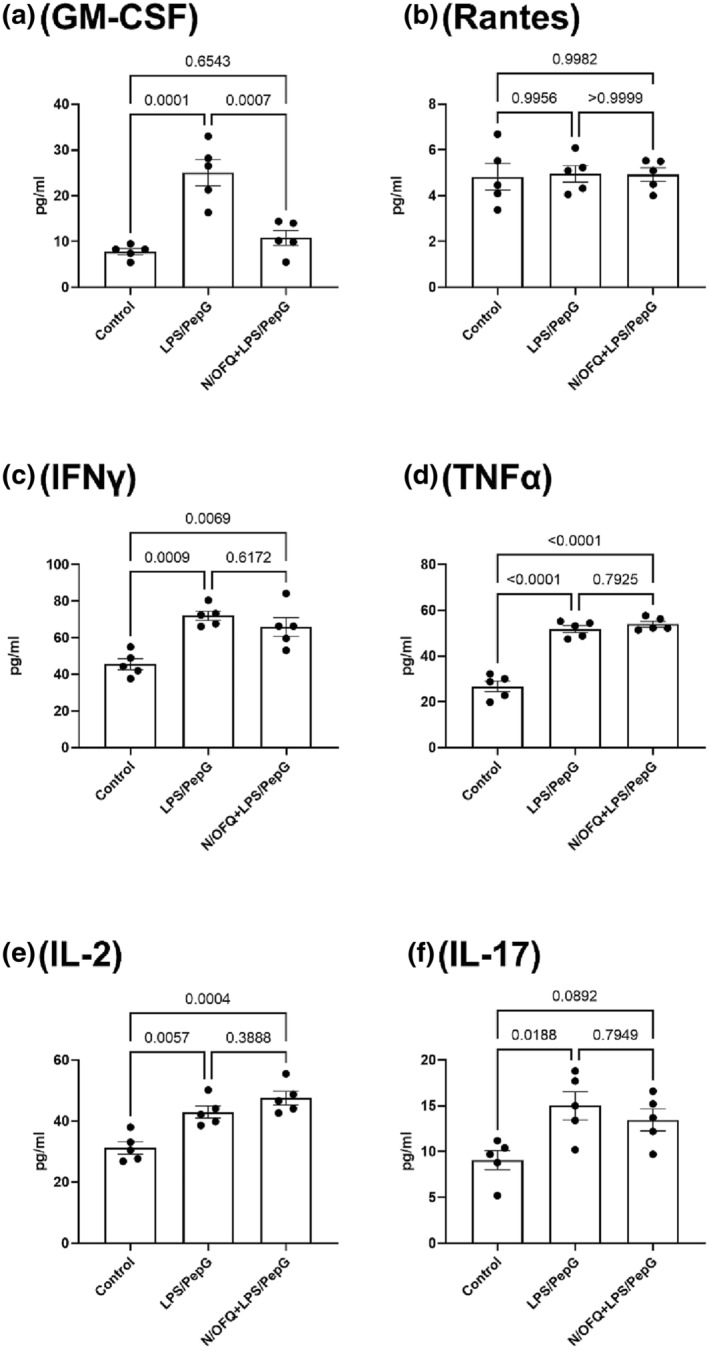
Cytokine/Chemokine release from T‐cells. N/OFQ (100 nM) and 100 ng·ml^−1^ LPS plus 20 μg·ml^−1^ PepG treatment was used. Data are the mean (±SEM) from five separate donors. Statistical analysis was carried out employing ANOVA. GM‐CST: *F*(2, 12) = 22.54:*P* < 0.0001; RANTES: *F*(2, 12) = 0.024:*P* = 0.976; TNF‐α: *F*(2, 12) = 73.46:*P* < 0.0001; IFN‐γ: *F*(2, 12) = 13.71:*P* = 0.0008; IL‐2: *F*(2, 12) = 16.05–:*P* = 0.0004; IL‐17: *F*(2, 12) = 5.875:*P* = 0.0166. Exact statistical significance (Sidak post hoc analysis) is shown.

Cytokine/Chemokine release assays were also performed on T‐cells, and six reached measurable threshold (GM‐CSF, RANTES, TNF‐α, IFN‐γ, IL‐2 and IL‐17). LPS (100 ng·ml^−1^)/PepG (20 μg·ml^−1^) stimulation increased the production and release of five of these detectable cytokines/Chemokines, with only RANTES not displaying increased release (Figure 9). The addition of 100‐nM N/OFQ reversed the production of GM‐CSF, but did not alter the increased release of the remaining four detectable cytokines/Chemokines.

## 4 DISCUSSION

In this study, we report the presence of NOP receptors on B‐cells and, following challenge with LPS/PepG (to mimic Gram‐negative and Gram‐positive polymicrobial sepsis), on T‐cells. In addition, B‐ and T‐cells contain immune‐reactive N/OFQ that can be released and measured in real time from single cells in a novel biosensor‐based assay. When present on the cell surface of naïve B‐cells, NOP receptor activation reduces migratory behaviour and in LPS/PepG treated cells reduces GM‐CSF production. When expression is induced by LPS/PepG in T‐cells, NOP receptor activation reduces migratory behaviour and GM‐CSF production. These data suggest both a constitutive and sepsis‐inducible N/OFQ‐NOP autocrine regulation of B‐ and T‐cell function, respectively.

Our suggestion that there is an N/OFQ‐NOP loop (constitutive and induced) deserves further comment. In this context, release of N/OFQ into the local environment will then act on expressed NOP receptors to then modulate their function. In our study, N/OFQ reduced migration. The importance of feedback/forward loops in adaptive immune response modelling has been reviewed (Rahman et al., 2018). One of the better known immune loops involves released ATP and is described as autocrine regulation. In this model, chemoattractants to B‐ and T‐cells draw cells to areas of need; there, they are stimulated to release ATP; this can be at immune cell junctions (immune ‘synapse’) or at other immune‐tissue contacts. Released ATP acts at P2Y2 receptors to amplify the original chemoattractant signal (Junger, 2011). Indeed, in our original paper describing single cell release of N/OFQ (Bird, Hebbes, et al., 2022), we provided the purinergic blocking protocol used in the current submission. Loops also occur in response to released cytokines that can affect the releasing cell or other immune cell types; an extreme example might occur in cytokine storm (Fajgenbaum & June, 2020). Concentrations are important to consider further and are discussed below; we have used 100‐nM N/OFQ. Local (immune ‘synapse’) concentrations in a zero‐flow system might get close to this, but in a physiological flow‐driven system, this might be a problem. In a series of unpublished experiments in freshly isolated human polymorphonuclear cells, we determined a pEC_50_ for N/OFQ inhibition of migration of ~10–50 pM, although the attractant was different (Al‐Hashimi et al., 2016).

We have determined the presence of NOP receptors on the surface of naïve and LPS/PepG treated B‐cells and in LPS/PepG treated T‐cells using our novel fluorescent ligand N/OFQ_ATTO594_ (Bird et al., 2018). That these were B‐ and T‐cells was confirmed with CD19‐pan B‐cell marker and CD3‐pan T‐cell marker expression. N/OFQ_ATTO594_ is an agonist at the NOP receptor and requires use at low temperatures to prevent internalisation; we have used it in recombinant systems, peripheral blood mononuclear cells (Bird et al., 2018), in vascular smooth muscle and endothelial cells (Bird et al., 2020) and mouse brain cell explants (Bird, McDonald, et al., 2022). In all cases, the binding was fully prevented by pre‐incubation with the NOP‐selective antagonist SB‐612111. In the present study, binding of N/OFQ_ATTO594_ was also fully prevented by SB‐612111 confirming ligand selectivity and NOP expression on the surface of B‐ and T‐cells. Moreover, this ligand bound in fixed and permeabilised cells and also in an SB‐612111 sensitive manner. A study from Arjomad et al. using end point PCR showed that CD19‐positive B‐cells (but not CD4‐positive T‐cells) expressed N/OFQ peptide transcripts. In addition, CD19‐positive B‐cells expressed NOP (the authors used old ORL‐1 terminology for NOP receptor in this paper), and there were very low but detectable levels of NOP in T‐cells (Arjomand et al., 2002; Easten et al., 2009; Waits et al., 2004). It should be noted that these data report mRNA and not biologically active protein.

With respect to the observation of N/OFQ_ATTO594_ binding in fixed‐permeabilised cells, we believe that B‐cells transcribe/translate then express cell surface receptors (this is measurable in permeabilised cells and on the surface of live cells); surface receptors are functionally active. This is independent of septic stimuli. In T‐cells, however, we believe we have transcription/translation but septic stimuli are required to traffic these receptors to the cell surface.

Circulating N/OFQ levels show marked variation (Barnes & Lambert, 2004); the source is presumed to be immune in nature, and we recently demonstrated release from single PBMCs using a novel bioassay (Bird, Hebbes, et al., 2022). We used CHO_hNOPgq/i5_ as biosensor cells where NOP is forced to couple to increased [Ca^2+^]_i_ that can be easily measured with FLUO‐4. If PBMCs are layered close to these biosensor cells and then stimulated with fMLP, released ‘material’ increased [Ca^2+^]_i_. When endogenous purinergic receptors are blocked (PBMCs can release ATP as noted above), the signal remained and was fully prevented by SB‐612111 confirming the released ‘material’ was N/OFQ. In the current study, we demonstrated the presence of N/OFQ by immunofluorescence in both B‐ and T‐cells (the fluorescent signal overlaps with that for NOP; N/OFQ_ATTO594_) and using the biosensor assay that this N/OFQ could be released in response to CXCL13 and IL‐4 challenge (Carlsen et al., 2002; Guo & Rothstein, 2013; Jenh et al., 2001; Mori et al., 2000) in B‐cells and CXCL12 and IL‐6 challenge (Nanki & Lipsky, 2001; Nish et al., 2014; Ticchioni et al., 2002; Weissenbach et al., 2004) in T‐cells. The stimulation also occurred in the presence of purinergic antagonists and the biosensor response could be fully prevented by SB‐612111. There are few other papers detailing release of N/OFQ, but Fiset et al. demonstrated in vitro release from populations of neutrophils using RIA as the detection technique (Fiset et al., 2003).

As noted, sepsis is a life‐threatening host response to infection leading to organ dysfunction (Singer et al., 2016). In the clinical setting, it is very difficult to know exactly which phase occurs at presentation, and this may lead to marked heterogeneity in clinical trial data. The coordination of immune responses in sepsis is complex with a significant role for polymorphonuclear and B‐ and T‐cells (Cao et al., 2019; Gustave et al., 2018; Hortová‐Kohoutková et al., 2021; Jensen et al., 2018; Kelly‐Scumpia et al., 2011; Monserrat et al., 2013); the latter being the focus of this study. We have used LPS/PepG as our in vitro sepsis mimic and show release of a panel of cytokines/chemokines. Indeed, we have shown elevated TNF‐α (~3×) in patients with a diagnosis of sepsis (Thompson et al., 2013), and this cytokine is also released (~×2) in vitro from B‐ and T‐cells stimulated with LPS/PepG. Whilst easy to administer, our sepsis cocktail should be considered a substitute for live bacteria, and future studies are warranted to more accurately mimic in vivo polymicrobial sepsis.

In B‐cells, GM‐CSF, eotaxin, TNF‐α, IFN‐γ, IL‐2, IL‐4, IL‐6 and IL‐12 (p40/p70) were detectable, and all were increased by LPS/PepG. GM‐CSF was inhibited by N/OFQ. In T‐cells, GM‐CSF, RANTES, TNF‐α, IFN‐γ, IL‐2 and IL‐17 were detectable. RANTES was not increased by LPS/PepG, and GM‐CSF was inhibited by N/OFQ. Several cytokines associated with sepsis, such as TNF‐α as noted above (Georgescu et al., 2020; Spooner et al., 1992) and IFN‐γ (Billi, 2020; Spiller et al., 2008), are increased with LPS/PepG treatment. Of the interleukins released by B‐cells, IL‐6 has demonstrated significant impact on mortality (Remick et al., 2005). Most importantly, the addition of N/OFQ does *not* alter release of cytokines associated with sepsis. GM‐CSF release is important for resistance to local infection and has been shown to restore cytokine secretion in monocytes, with many clinical studies demonstrating GM‐CSF as a potential treatment for sepsis (Mu et al., 2021). The inhibition of GM‐CSF by N/OFQ would therefore appear to act as a pro‐sepsis response. That only GM‐CSF is consistently inhibited is worthy of further note, although we have no concrete explanation for this finding. We might have expected changes in other cytokines but this may be model related.

In a small clinical study in ICU patients, we measured immune‐reactive N/OFQ (by RIA); this was increased in patients who died compared with those who survived (Williams et al., 2008). In a larger study, we measured N/OFQ (again by RIA) in patients on the first day and second day of admission to ICU and compared this to plasma concentrations when the patients had recovered; N/OFQ was increased at both time points. We also reported reduced NOP mRNA that we suggested was used to translate into receptor protein (Thompson et al., 2013). At the time, we did not have N/OFQ_ATTO594_ to track surface receptors, and there was not sufficient material for a traditional radioligand binding experiment. Moreover, we were not confident at the time of selectivity of available receptor antibodies for use with fixed cells or in Western blotting. N/OFQ was shown to be detrimental to outcome in a caecal‐ligation and puncture model of peritoneal sepsis in rats. In this model, Carvalho et al. showed that systemic administration of N/OFQ (agonist) worsened and that UFP‐101 (NOP antagonist) (Carvalho et al., 2008) improved survival. In this model, systemic N/OFQ also worsened the increase in TNF‐α, IL‐1β and MCP‐1, and UFP‐101 blunted the increase in these same cytokines (Carvalho et al., 2008). It should be borne in mind that, to date, this is the only animal study. In non‐immune cells, an LPS‐induced up‐regulation of N/OFQ peptide expression has also been shown in mouse sensory neurones; NOP was not studied (Acosta & Davies, 2008).

In order to examine B‐ and T‐cell function in response to NOP activation, we used Bodipy_488_ dye and transwell inserts to track migration. B‐cell migration was measured towards the chemoattractants, CXCL13 and IL‐4 whilst migration in T‐cells was towards CXCL12 and IL‐6. The pattern of migration in these series of experiments is complex. In B‐cells, LPS/PepG does not increase migration, whereas in T‐cells, there is more than a doubling of migration. The response in B‐cells is supported by similar findings in mice (Moon et al., 2012). In naive B‐cells, N/OFQ tended towards reduced migration in an SB‐612111 sensitive fashion, and in T‐cells, there is no NOP‐driven response, consistent with a lack of receptors. In the presence of LPS/PepG, the N/OFQ mediated inhibition of migration in B‐cells is lost, but there is a reduction to SB‐612111. In T‐cells treated with LPS/PepG (induced expression), N/OFQ now inhibits migration in an SB‐612111 sensitive manner; this mirrors the response in naive B‐cells.

In B‐cells, the trend towards migration inhibition with constitutive NOP receptor expression is lost with LPS/PepG treatment. This is hard to explain in the face of T‐cells requiring LPS/PepG treatment to translate NOP receptor mRNA into active protein and then inhibit migration. In B‐cells, whilst there is no increase in migration with LPS/PepG, there is increased levels of cytokines. It is tempting to speculate that these cytokines and LPS/PepG treatment might be detrimental to cell survival but we have little evidence to support this.

There are some data showing that N/OFQ (pM), reduced antibody formation to sheep red blood cells in a plaque assay (Anton et al., 2010). B‐ and T‐cell interaction is important in the innate immune response and increased migration to LPS/PepG may direct these immune cells to the site(s) of infection. It is not unreasonable to suggest that when resident they could release N/OFQ to activate NOP and retain them at the infection site. Clearly, these are suppositions based on in vitro data from single immune cell types that require further in vivo study. Examining N/OFQ‐NOP responses in a recreated immune interactome is worthy of further study. More difficult to explain is the SB‐612111 response in LPS/PepG treated B‐ and T‐cells. There is no primary response to N/OFQ in B‐cells yet the antagonist produced a small but significant response alone or with exogenous N/OFQ present. Non‐NOP receptor actions of SB‐612111 are not well described but as this occurs in both B‐ and T‐cells, it is likely to be a non‐specific effect of the antagonist. As this occurs in LPS/PepG treated conditions, it is possible that SB‐612111 interacts with TLR receptors as noted for some classical opioids (TLR4) (Giakomidi et al., 2022; Hutchinson et al., 2012), and there is evidence for NOP receptor interaction with TLR receptors, but no data for SB‐612111 (Zhang et al., 2022).

**FIGURE 10:**
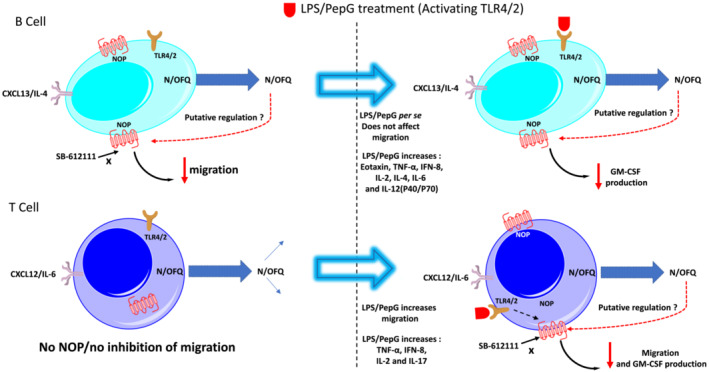
Constitutive N/OFQ‐NOP receptor expression and regulation of function in B‐cells and LPS/PepG inducible expression and regulation of function in T‐cells. NOP receptors and N/OFQ are constitutively expressed in B‐cells where stimulation with CXCL13 and IL‐4 increases N/OFQ release. This stimulation also promoted migration that can be inhibited by N/OFQ via a putative autocrine feedback onto NOP receptors*. LPS/PepG treatment per se does not affect migration (to the chemoattractants), but the LPS/PepG stimulated increase in GM‐CSF is reduced by N/OFQ. T‐cells do not express NOP receptors constitutively but they do express N/OFQ that can be released by CXCL12 and IL‐6. LPS/PepG treatment per se increased migration (to the chemoattractants), and this can be inhibited by N/OFQ via a putative feedback onto NOP receptors*. LPS/PepG induced increase in GM‐CSF release was also inhibited by N/OFQ. *Putative feedback loops are based on the addition of exogenous (100 nM) N/OFQ. LPS/PepG activates TLR4/2.

In summary, our data are suggestive of constitutive N/OFQ‐NOP autocrine regulation in isolated human B‐cells and a similar but LPS/PepG inducible regulation in isolated human T‐cells. These NOP receptors variably inhibit migration and reduce GM‐CSF production (Figure 10). In general, this release of N/OFQ and NOP activation has the potential to lead to detrimental outcome. It is tempting to suggest that use of NOP antagonists in sepsis represents a potential adjuvant for this syndrome with few current treatment options available. LY2940094 is a potent and selective NOP antagonist (Ferrari et al., 2020) currently in development for other disease indications (NCT03193398 and NCT03608371); its effects in sepsis are unknown (Witkin et al., 2016; Witkin et al., 2019).

## AUTHOR CONTRIBUTIONS

Mark F. Bird and Christopher P. Hebbes were responsible for primary data collection and analysis and writing the paper. Jonathon Mark Willets was responsible for obtaining funding and writing the paper. Jonathan P. Thompson was responsible for the analysis and writing the paper. Remo. Guerrini and Girolamo Calo were responsible for provision of fluorescent probe, analysis and writing the paper. Anushuya Tamang was responsible for cytokine/chemokine data collection‐analysis and writing the paper. David G. Lambert was responsible for obtaining funding, analysis and writing the paper. All authors approved the final version.

## CONFLICT OF INTEREST STATEMENT

DGL is a scientific adviser to Cellomatics Biosciences Ltd.

## DECLARATION OF TRANSPARENCY AND SCIENTIFIC RIGOUR

This Declaration acknowledges that this paper adheres to the principles for transparent reporting and scientific rigour of preclinical research as stated in the *BJP* guidelines for Design and Analysis, and as recommended by funding agencies, publishers and other organisations engaged with supporting research.

## Supporting information


**Appendix S1.** Supporting Information

## Data Availability

All data are available in the submission or the supporting information.
