# Supplementary material for: In vitro sepsis up‐regulates Nociceptin/Orphanin FQ receptor expression and function on human T‐ but not B‐cells
Source: Br J Pharmacol. 2023 May 11;180(17):2298–314. doi: 10.1111/bph.16088 (PMC10953342; doi:10.1111/bph.16088)
Supplement: Supplementary file 1 — Appendix S1. Supporting Information [file BPH-180-2298-s001.pdf]

In vitro sepsis upregulates Nociceptin/Orphanin FQ receptor expression and function on human T- but not B-cells.

[1]Bird MF, [1]Hebbes CP, [2]Tamang A, [3]Willets J, [1]Thompson JP, [4]Guerrini R, [5]Calo G and [1*]Lambert DG[1*].

[1]Departments of Cardiovascular Sciences and [3]Molecular and Cell Biology, University of Leicester, Anaesthesia, Critical Care and Pain Management, Hodgkin Building, Leicester, LE1 9HN. UK.

[2]Cellomatics Biosciences Ltd, 10 Colwick Quays Business Park, Road No 2, Colwick, Nottingham, NG4 2JY, UK

[4]Department of Chemical, Pharmaceutical and Agricultural Sciences, University of Ferrara, 44121 Ferrara, Italy.

[5]Department of Pharmaceutical and Pharmacological Sciences, University of Padova, 35131 Padova, Italy.

Appendix

1. Recombinant NOP as a control for N/OFQATTO594 binding:

In Figure 1, we demonstrate binding of N/OFQ$_{ATTO594}$ to recombinant NOP expressed in CHO cells as an exemplar high expression system. This visual comparator indicates the significantly lower levels of expression in B- and T-cells in the main paper.

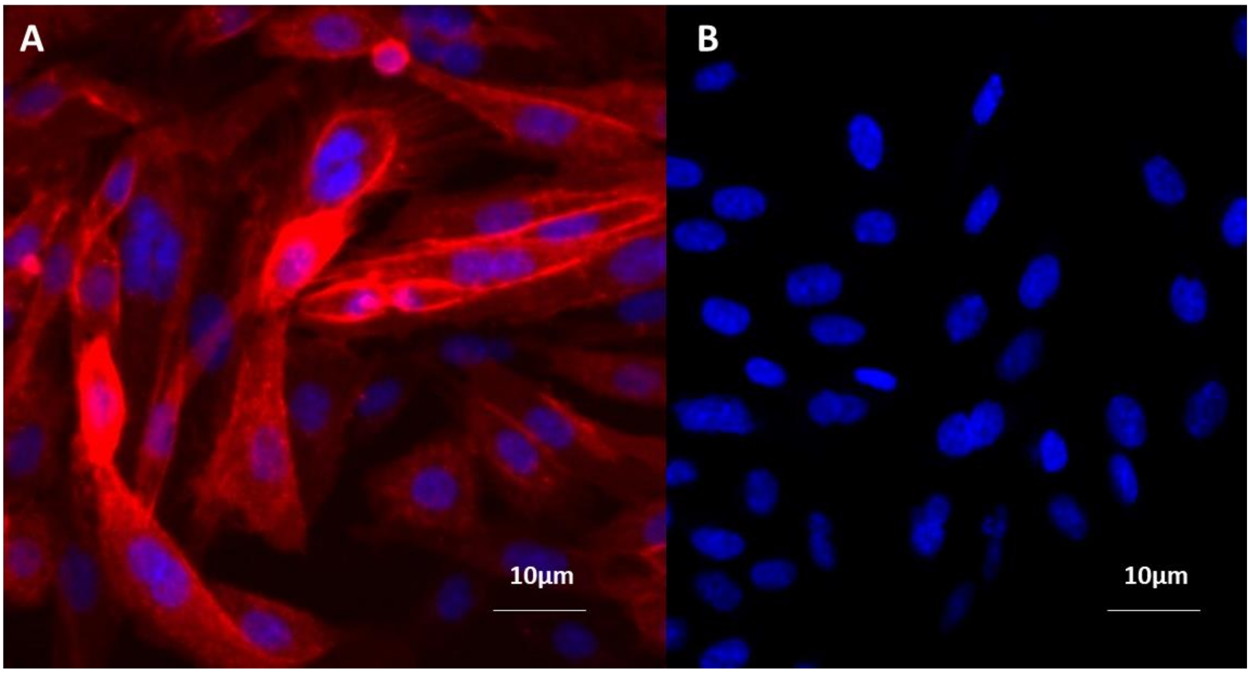

Figure 1: (A) A representative image demonstrating binding of 100nM N/OFQ$_{ATTO594}$ in CHO$_{hNOP}$ cells. These cells express with high levels (~1pm/mgp) of the NOP receptor. (B) A representative image showing 10µM SB-62111 inhibition of N/OFQ$_{ATTO594}$ binding confirming selectivity. Nuclei are stained with Hoechts dye (Blue).

2. Immunofluorescence controls:

These are shown for B-cells (**Figure 2**) and T-cells (**Figure 3**) Cells were labelled with Brilliant violet™ anti-CD19 (B-cells) or Brilliant violet™ anti-CD3 (T-cells), anti-N/OFQ (non-fluorescent) antibody and 10µM SB-612111, before being incubated with the desired concentrations of anti-N/OFQ$_{FITC}$ (1:1000) and N/OFQ$_{ATTO594}$ (100nM), with measurements taken at 420nm (**Figure 2A)**, 488nm (**Figure 2B**) and 594nm (**Figure 2C**) excitation wavelengths. In B cells, the anti-CD19 marker fluoresced (**Figure 2A**), while no excitation emission was seen in either the green (**Figure 2B**) or red (**Figure 2C**) channels. In corresponding T cell experiments, fluorescence was seen in the blue channel (**Figure 3A**), but not in the green (**Figure 3B**) or red channels (**Figure 3C**).

To determine N/OFQ$_{FITC}$ selectivity, cells were incubated with anti-N/OFQ$_{FITC}$ antibody in combination with Brilliant violet™ anti-CD19 in B cells or Brilliant violet™ anti-CD3 in T cells and 10µM SB-612111 before addition of 100nM N/OFQ$_{ATTO594}$. In these experiments, fluorescence was detected in the blue channel (**Figure 2D, Figure 3D**) and the green channel (**Figure 2E, Figure 3E**). No fluorescence was detected in the red channel for either B cells (**Figure 2F**) or T cells (**Figure 3F**).

The final control experiments combined Brilliant violet™ anti-CD19 in B cells or Brilliant violet™ anti-CD3 in T cells with N/OFQ$_{ATTO594}$. Cells were preincubated with anti-N/OFQ antibody before addition of anti-N/OFQ$_{FITC}$ antibody. In these experiments, fluorescence was detected in the blue channel (**Figure 2G, Figure 3G**) and red channel (**Figure 2I, Figure 3I**) but not in the green channel (**Figure 2H, Figure 3H**).

These data demonstrate (1) marker selectivity and (2) no "leak" of fluorescence into other channels.

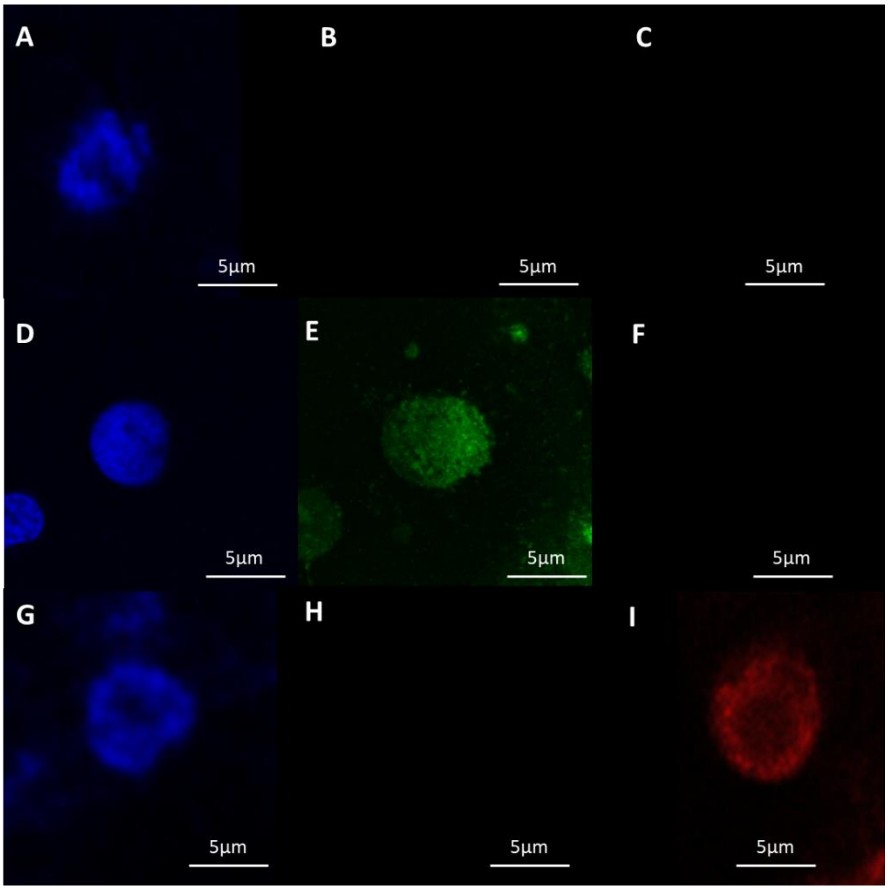

Figure 2: A series of experiments using fluorescent markers to determine specificity of binding of chosen fluorescent ligands in B cells. Data are representative of 5 separate experiments.

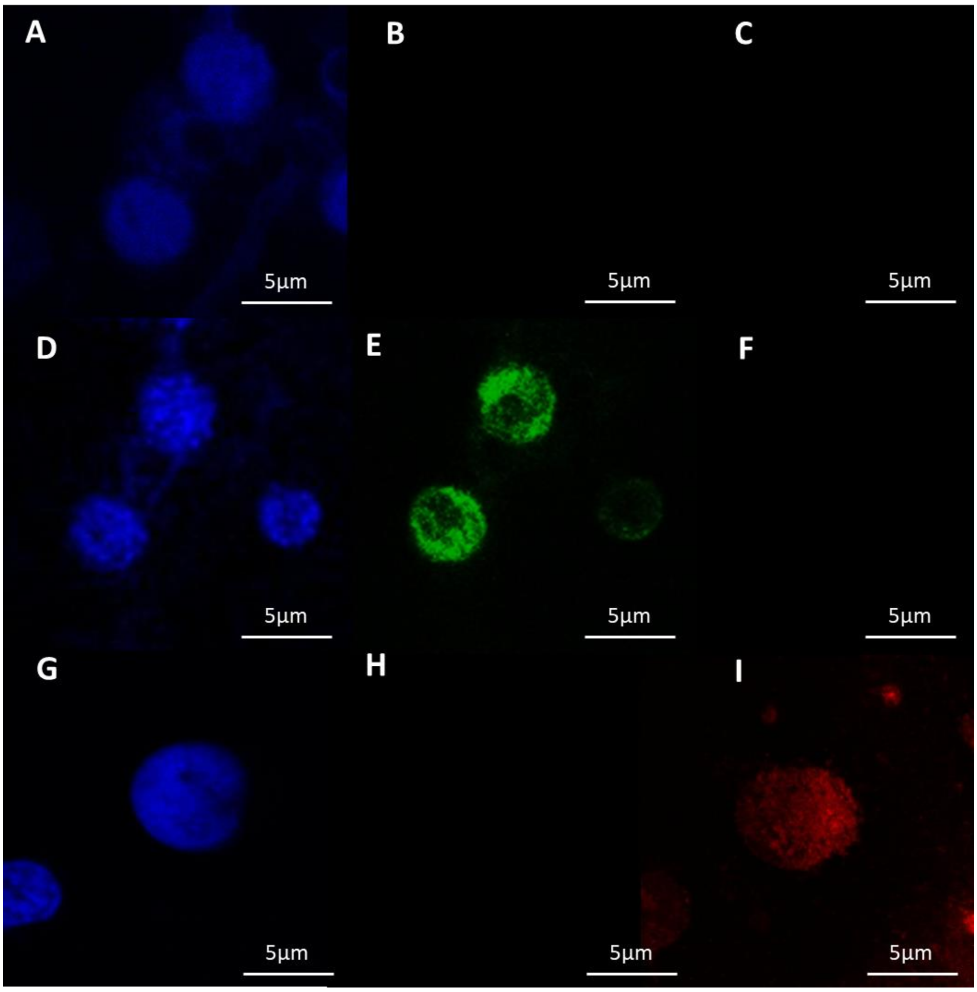

Figure 3: A series of experiments using fluorescent markers to determine specificity of binding of chosen fluorescent ligands in T-cells. Data are representative of 5 separate experiments.
